# Mechanical effect of reconstructed shapes of autologous ossicles on middle ear acoustic transmission

**DOI:** 10.3389/fbioe.2023.1204972

**Published:** 2023-06-22

**Authors:** Takumi Asakura, Ryuya Ito, Motoki Hirabayashi, Sho Kurihara, Yuta Kurashina

**Affiliations:** ^1^ Department of Mechanical Engineering, Faculty of Science and Engineering, Tokyo University of Science, Chiba, Japan; ^2^ Hitachi Corporation, Ibaraki, Japan; ^3^ Department of Otorhinolaryngology, The Jikei University School of Medicine, Tokyo, Japan; ^4^ Division of Regenerative Medicine, The Jikei University School of Medicine, Tokyo, Japan; ^5^ Division of Advanced Mechanical Systems Engineering, Institute of Engineering, Tokyo University of Agriculture and Technology, Tokyo, Japan

**Keywords:** vibroacoustic analysis, finite element method, conductive hearing loss, artificial middle-ear prosthesis, autologous ossicle

## Abstract

Conductive hearing loss is caused by a variety of defects, such as chronic otitis media, osteosclerosis, and malformation of the ossicles. In such cases, the defective bones of the middle ear are often surgically reconstructed using artificial ossicles to increase the hearing ability. However, in some cases, the surgical procedure does not result in increased hearing, especially in a difficult case, for example, when only the footplate of the stapes remains and all of the other bones are destroyed. Herein, the appropriate shapes of the reconstructed autologous ossicles, which are suitable for various types of middle-ear defects, can be determined by adopting an updating calculation based on a method that combines numerical prediction of the vibroacoustic transmission and optimization. In this study, the vibroacoustic transmission characteristics were calculated for bone models of the human middle ear by using the finite element method (FEM), after which Bayesian optimization (BO) was applied. The effect of the shape of artificial autologous ossicles on the acoustic transmission characteristics of the middle ear was investigated with the combined FEM and BO method. The results suggested that the volume of the artificial autologous ossicles especially has a great influence on the numerically obtained hearing levels.

## Introduction

Conductive hearing loss occurs when the middle ear is damaged by various ear diseases, such as chronic otitis media, osteosclerosis, and ossicle malformation. In such a case, the tympanoplasty operation is often carried out to reconstruct the damaged ossicular chain. In the ossicular chain reconstruction, an artificial ossicle (AO) is installed into the middle ear. The prognosis of hearing recovery is affected by AO variations, including the material characteristics (Jahnke and Plester, 1981; Grote et al., 1986; Beutner and Hüttenbrink, 2011; Shah et al., 2013; Jiang et al., 2017; Lahlou et al., 2018; Siddappa et al., 2019); mounting method (Fleischer, 1991); and the shapes of specific geometries, such as the S-shaped prosthesis (Seivur et al., 2022), ball-type structure (Stoppe et al., 2017; Ren et al., 2020), C-shaped Shape memory alloys (SMA) prosthesis (Latalski et al., 2017), the partial ossicular replacement prosthesis (PORP), and the total ossicular replacement prosthesis (TORP) (Kelly et al., 2003). It has been found that some of these materials with low biocompatibility tend to be rejected by the body when they are placed in the middle ear. For example, AOs composed of titanium or hydroxyapatite have a relatively high rate of rejection (Siddappa et al., 2019). However, it is possible to realize AOs with high biocompatibility by using autologous bone, such as the auricle or nasal septal cartilage of the patient, or by diverting unnecessary autologous residual ossicles. Autogenous bone grafting is a beneficial choice for middle-ear reconstruction because it is easy to obtain at the surgical site, is non-toxic, shows a low rate of rejection, and gives good hearing gain (Siddappa et al., 2019). The influence of the geometry (Koike et al., 2002) and physical properties (Greef et al., 2017) of AOs has been numerically studied, but there are no examples of parametric verification of the influence of their geometry from the standpoint of mechanical vibration. In the abovementioned studies, numerical analysis techniques based on the finite element method (FEM) have been applied. Because the FEM is a powerful tool, many kinds of engineering studies have used this method to clarify the physical characteristics inside biological bodies (Ashwani et al., 2014; Chang et al., 2018; Gao et al., 2021; Zhao et al., 2021). Regarding the application of the FEM to the field of otolaryngology, some numerical investigations can also be seen related to the human upper airway (Luo et al., 2017), the stress distribution in human mandibles (Hassan et al., 2018), vibroacoustic transmission through the human middle ear (Koike et al., 2002), the vocal fold vibration in normal phonation (Jiang et al., 1998), the deformation of the nasal model (Manuel et al., 2017), and the mechanism of acoustic transmission through the ears of animals (Funnel and Laszlo, 1978; Hoffstetter et al., 2011; Maftoon et al., 2015).

Regarding the FEM analysis in the field of audiology, in the 1970s, the first FEM model of the cat’s tympanic membrane (TM) was implemented by Funnell and Laszlo (Funnel and Laszlo, 1978). Afterward, various FEM models were investigated to study the dynamic behavior of the middle ear. In Wada et al. (Wada et al., 1997), an FEM model of the human middle ear was first implemented. The validity of the model was then extended (Wada et al., 1996) to new modes, including anterior mallear and posterior incudal ligaments, tensor tympani and posterior stapedial tendons, the middle-ear cavity as a rectangular solid, and the ear canal (EC) as a rigid tube, respectively. Then, Koike et al. further modified the above model by adding ligaments, tendons, the incudostapedial joint, the external EC, and middle-ear cavities (Koike et al., 2002). Wang et al. considered the nonlinearities of the materials by introducing the hyperelastic Mooney-Rivling properties (Wang et al., 2007). Then, in 2009, Homma et al. (2009) revealed that the dominant mode of the ossicular chain under air-conducted excitation is the first mode that is characterized by hinging ossicular motion, whereas the dominant mode under bone-conducted excitation is the second mode that is characterized by pivoting ossicular motion. Then, Wang and Gan (2016) used a 3D FEM model of the chinchilla TM and middle ear to compute the distribution of stress in the TM and the TM displacement with impulse pressure waves. Recently, Gyliene et al. investigated the mechanical effect of the incus (Gyliene et al., 2010). In addition, Tu et al. (2017) simulated 30 ears using FEM models generated using the scanning data obtained by computed tomography and clarified that the FEM can be effectively used as a surgical assessment tool in clinics.

However, the FEM takes a relatively large amount of computational time to calculate a single condition. Moreover, the computation time increases with the number of conditions when searching for the optimum condition based on the results of many calculations. In one research case (Gentil et al., 2015), the FEM was used to search for optimal conditions for the thickness of an AO by comparing among different cases, but given the computational time, this method is not suitable for a situation where optimal conditions are searched from a large number of conditions. Therefore, it is necessary to optimize the computational conditions as much as possible and conduct an efficient numerical search. The search for optimal experimental conditions involves determining the preferred method of design of experiment (DOE), and then conducting the analysis iteratively based on the information obtained from the DOE. In such cases, methods like random search (Rafajlowicz, 1998), grid search (Pontes et al., 2016), Bayesian optimization (BO) (Močkus, 1974), and genetic algorithms (Holland, 1975) are available, but when the target of optimization has a 3D shape, the number of possible conditions for the shape has a wide range, and the computation time required for the search can be a problem. To avoid a high computational cost, it is desirable to use the most efficient method possible. While the methods of random and grid search are exhaustive, they are also brute-force estimators (Alsharef et al., 2022), and thus are not very desirable. From the viewpoints of computational efficiency, evolutionary algorithms, including genetic algorithms, generally require thousands of evaluations to be competitive with more sample-efficient methods such as BO (Turner et al., 2021). BO has been investigated from the 1970s (Močkus, 1974) and has been widely used as a method of DOE in various fields. Optimization with this method has also been used in the hearing field (Korzepa et al., 2020; Milazzo et al., 2020; Balling et al., 2021; Marco and Bert, 2021; Dargah et al., 2023), especially for the purpose of hearing aid research. Milazzo et al. have proposed a novel optimization scheme and have shown the validity of the method to obtain highly flexible optimized results that can be built on such a way as 3D printings. In this case study, very precise shape design is possible through non-parametric topology optimization, which has a large impact on the design process of TORPs and PORPS. On the other hand, as mentioned above, when using autologous bone, the AO needs to be cut out manually by the operator. So, it is not practical to cut out complex shapes. In such cases, it is desirable to optimize parameters related to shapes such as relatively simple column structures by using a parametric optimization scheme.

In such a parametrical optimization process, iterative calculations are performed based on some methods, requiring an efficient scheme to describe the target shape with simple parameters. Various kinds of shape descriptors, such as the method using Fourier series (Kyung et al., 1987; Burger and Burge, 2013), have been proposed. While this method can be applied to any shape as long as it is a closed shape, superposition of higher-order coefficients to represent such angular shapes requires a convergence calculation with a relatively large amount of iteration time. However, for optimization of structural shapes, the basis vector method (BVM), which is based on several basic shapes and resynthesizes the original shape by superposition of these shapes, has been proposed and recently used in the structural field (Wu et al., 2020). This method has been used since the 1980s (Belegundu and Rajan, 1998; Vanderplaast and Miura, 2012), and it is often paired with the FEM. Because a basic shape can be freely selected for the model of interest, it is expected to be less computationally expensive and simpler than methods that approximate the original shape by functions such as Fourier series. Therefore, it is considered suitable for optimization of relatively simple shapes, such as the AO of this study.

In this study, to estimate the hearing restoration effect prior to the tympanoplasty operation, the effect of the shape of the reconstructed AO was investigated through a numerical approach using FEM analysis. By this approach, it is possible to understand the vibroacoustic mechanism of the acoustics transmission through an ossicular prosthesis. Then, to fully understand the effect of the AO shape by efficiently choosing the calculation condition of its shape, an optimization technique using the coupling method of BO and BVM (BO-BVM) was additionally adopted. First, a FEM simulation was carried out to investigate the frequency response of the stapes footplate, and the numerical results were validated through comparison with former experimental and numerical results. Then, operation models of tympanoplasty were targeted, and the effect of AO shapes on the acoustic transmission characteristics was obtained by an updating calculation based on BO-BVM. The results indicated that especially the volume of the artificial ossicular prostheses has a great influence on the numerically obtained hearing levels.

## Methods

This section describes the overall numerical scheme of the FEM analysis and the shape updating scheme for the artificial prosthesis using the combined BO-BVM method.

### FEM analysis

To simulate the sound transmission characteristics via the ossicular chain, the following equation of motion was used in the FEM:

$$\left(\left[\begin{array}{cc} K_{S} & -C\\ 0 & K_{A} \end{array}\right]+j\omega \left[\begin{array}{c} \begin{array}{cc} D_{S} & 0 \end{array}\\ \begin{array}{cc} 0 & D_{A} \end{array} \end{array}\right]-\omega^{2}\left[\begin{array}{cc} M_{S} & 0\\ C^{T} & M_{A} \end{array}\right]\right)\left\{\begin{array}{c} x\\ p \end{array}\right\}=\left\{\begin{array}{c} f_{S}\\ f_{A} \end{array}\right\}$$ (1)

where *K*, *D*, *M*, and *C* indicate stiffness, damping, mass and coupling matrices, respectively, and *x*, *p*, and *f* indicate the vectors of displacement, sound pressure and force, respectively. The structural components are denoted by subscript S and the acoustic components by subscript A. The analysis was performed using Simcenter Nastran from Siemens.

### Vibroacoustic FEM model of a healthy middle ear

**FIGURE 1:**
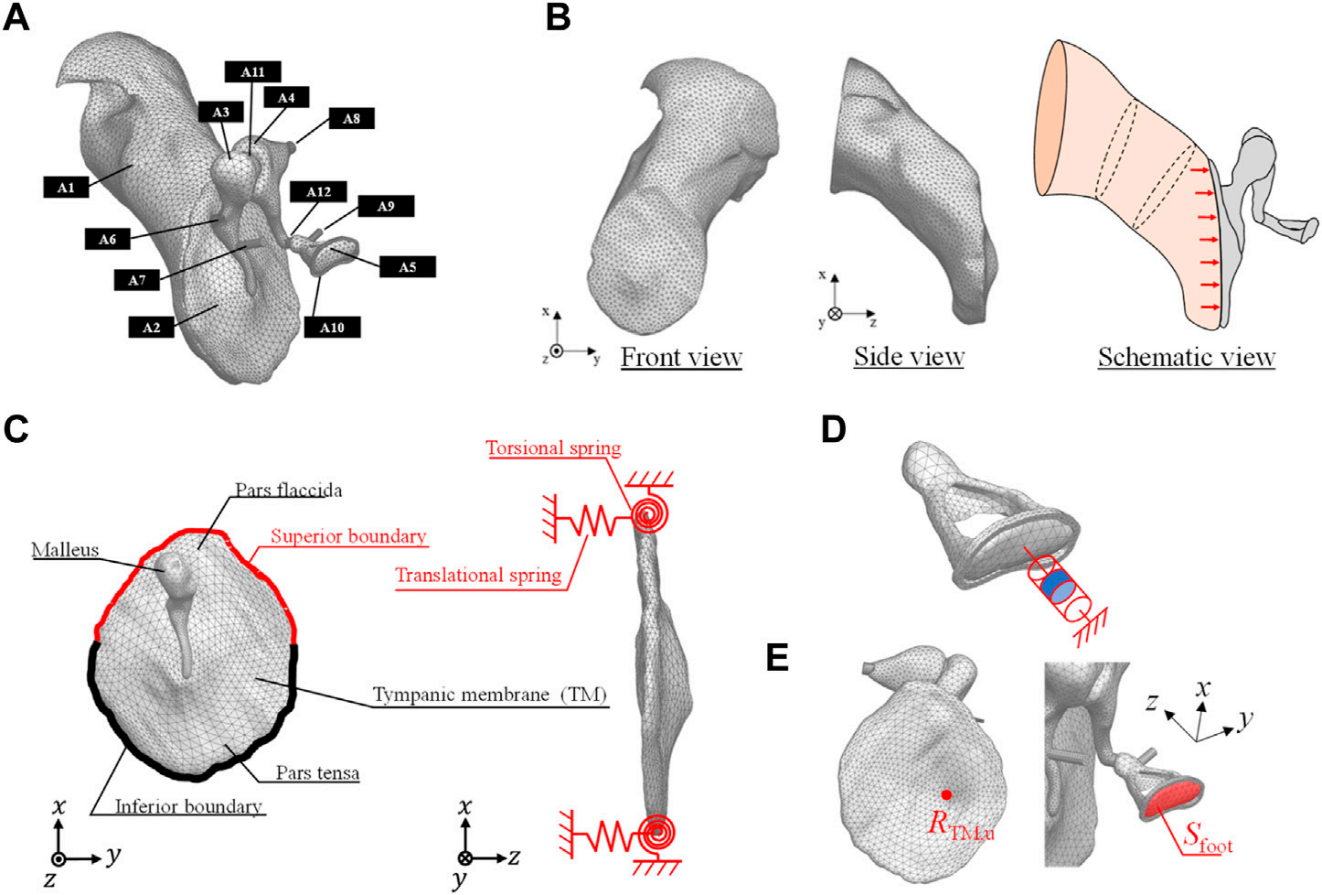
**(A)** FEM model of a human right ear that includes the (A1) ear canal (EC) (A2) tympanic membrane (TM) (A3) malleus (A4) incus (A5) stapes (A6) anterior malleolar ligament (A7) tensor tympanic tendon (A8) posterior incudal ligament (A9) stapedial tendon (A10) stapedial annular ligament (A11) incudomalleolar joint, and (A12) incudostapedial joint. **(B)** FEM model of the EC in the front and side views with its schematic view excited by acoustic load on the TM. **(C)** Boundary conditions of the vibration field of the TM pars flaccida and pars tensa. **(D)** Numerical model of the viscoelastic characteristics of the stapes footplate influenced by the resistance of the inner ear lymph fluid. **(E)** Receiving point of R_TM,u_ on the TM and the surface area of the footplate.

The three-dimensional model of a healthy human middle ear used in this analysis is shown in Figure 1A. The EC, TM, and ossicles were modeled using a 3D model generated by micro-computed tomography (Sieber et al., 2019) and provided as STL files. The model was discretized by tetrahedral quadratic elements by the Simcenter Nastran, and used in the FEM simulation. The vibration model of the TM and ossicles and the acoustic model of the sound field inside the EC were coupled at the boundary surface between the TM and sound field of the EC. This coupling is naturally considered by solving Eq. 1. In this 3D model, the tendons, ligaments and joints attached to each of the ossicles were simply modeled as cylinders and connected to the middle ear model as shown in the figure. Each of the ossicles were coupled by (A11) incudomalleolar and (A12) incudostapedial joints shown in Figure 1. To realize continuity between the ossicles and the joint parts, the displacements of the discrete elements on each of the ossicles and joint parts are made to be identical in the simulation. Then, these joint parts work quite flexibly due to the lower Young’s modulus compared to those of ossicular bones, as shown in Table 1. The annular ligament surrounding the stapes was modeled to be 0.16 mm wide and 0.20 mm thick along the base plate of the stapes, as done in Koike et al. (2002). The material conditions for each part were all considered as isotropic elastic materials by assuming that the displacement with respect to the model dimensions was sufficiently small and that the nonlinearity of the tendons and ligaments could be neglected because it has been reported that the displacement of the middle ear is on the order of nanometers. The physical properties adopted in this study are summarized in Table 1. The values of density and elastic modulus were taken from Koike et al. (2002).

**TABLE 1:** Material properties used to create the FE model.

	Density (kg/m^3^) [14]	Young’s modulus (MPa) [14]	Poisson’s ratio (−)	Loss factor (−)
Tympanic membrane (tensa)	1,200	33.4	0.3	0.5
Tympanic membrane (flaccida)	1,200	11.1		0.5
Malleus	2,390	1.20 × 10^4^		0.01 [30]
Incus	2,150	1.20 × 10^4^		0.01 [30]
Stapes	2,200	1.20 × 10^4^		0.01 [30]
Anterior mallear ligament	2,500	21		0.5
Tensor tympani muscle	2,500	2.6		0.5
Posterior incudal ligament	2,500	0.65		0.5
Incudostapedial joint	2,500	6.0		0.5
Stapedius muscle	2,500	0.52		0.5

As for the loss factor of the middle ear, a previous study (Homma et al., 2009) gives a value of around 0.15 based on measurements on the temporal bone, while another study (Koike et al., 2002) gives a value of around 0.1 for the ligaments based on Rayleigh damping at 1 kHz, but a value as high as 3.0 is given for the incudostapedial joints and all other sites, with variation between studies. Moreover, none of the other studies (Aibara et al., 2001; Gan et al., 2004; Fay et al., 2006; Sim et al., 2007) have shown a clear indication. This is due to the fact that the energy loss in the ear ossicles includes the damping effects of the TM, ossicles, and stapes footplate connected to the lymphatic fluid in the inner ear (which are difficult to identify one by one). Also the existing literature (Homma et al., 2009) has identified the total energy loss and a loss factor tuned by comparing the simulative and experimental results is given on average for all components in the ear ossicles. Thus, the loss factor in vibration analysis is difficult to give, while the output level at the peak frequency at each mode frequency is affected by it, so tuning is essential, even if its physical meaning is questionable. In the present study, 0.5 was given on average to elements other than the ear ossicles, with reference to the values given in the literature (Koike et al., 2002; Homma et al., 2009). Among them, only the loss factor for the ear ossicles, which are considered to be relatively hard and have relatively low damping characteristics, was given a value of 0.01, as done previously (Homma et al., 2009).

### FEM model of the sound field inside the EC

Figure 1B shows the FEM model of the EC. This model was discretized by linear tetrahedral elements. As the end surface on the tympanic side is in contact with the TM surface, the element size of the surface adjacent to the TM was made to be the same as that of the FEM model of the TM. Next, the schematic diagram of the boundary conditions of the sound field inside the EC is shown in the same figure. One end of the EC is closed by the TM, while the other end is open because it is connected to the auricle. Therefore, not only does sound enter from the outside, but the sound that enters is reflected by the TM, passes through the EC, and is radiated to the exterior sound field again. To represent this, a perfect sound absorption condition was set at the auricular end of the EC, to absorb the sound reflected by the TM. To obtain comparable data with respect to the previously measured data, the sound source was set to be a plane source with a constant sound pressure of 80 dBSPL against the TM surface, as ordinarily done in previous studies.

### Boundary conditions in the vibration FEM model

Figure 1C shows a schematic of the TM. The TM is fixed to the EC by the tympanic ring, which is made of soft biological tissue. In a previous study (Koike et al., 2002), the boundary condition of the tympanic ring was assumed to be working as an elastically supported boundary, which was simulated by providing linear and rotational springs. Therefore, in this study, this modeling method was adopted, and the same spring constants for the linear and rotational springs used in reference (Koike et al., 2002) were assigned, as shown in Table 2. Next, Figure 1D shows the method of modeling the viscoelastic characteristics of the stapes footplate using the damper. The stapes is connected to the cochlea of the inner ear, which is filled with lymphatic fluid. In a previous study (Møller, 1965), the results of impedance measurements of a cat’s middle ear clarified that the damping effect caused in the footplate is mostly due to the cochlea. In addition, cochlear impedance measurements of the human temporal bone have also shown that the damping effect is dominant in the input impedance of the cochlea, especially at around 1 kHz (Aritomo and Goode, 1987). Therefore, in the present analysis, as in previous studies (Koike et al., 1996), the cochlea was assumed to exert only a damping effect on middle-ear vibrations, and a viscous damper was connected to the footplate. The loading of the cochlea on the stapes footplate was assumed to be 0.62 N s/m, making the impedance of the cochlea equivalent to 35 GV (Zwislocki, 1965).

**TABLE 2:** Spring coefficients of the linear and torsional springs modeling the elastically supported boundary condition of the TM.

Component	Linear spring (N/m∙m)	Torsional spring (N∙m/m)
Tympanic ring (Superior)	3.0×10^3^	3.0×10^−5^
Tympanic ring (Inferior)	1.5×10^5^	1.0×10^−4^

### Vibroacoustic FEM model of the middle ear with an artificial prosthesis

In this study, a basic type of healthy middle ear and four types of reconstructed middle ears selected based on Wullstein’s typology (Wüllstein, 1965), for a total of five patterns, were investigated. The four types of reconstructed middle ears are as follows: 1) Type III_c_, which interposes a short columella between the stapes head and the TM; 2) Type III_i-M_, which interposes a short columella between the stapes head and the manubrium; 3) Type IV_c_, which interposes a relatively long columella between the stapes footplate and the TM; and 4) Type IV_i-M_, which also interposes a relatively long columella between the stapes footplate and the manubrium. Note that the latter Types IV_c_ and IV_i-M_ are mainly used when the stapes superstructure is missing (Bahmad et al., 2022).

**FIGURE 2:**
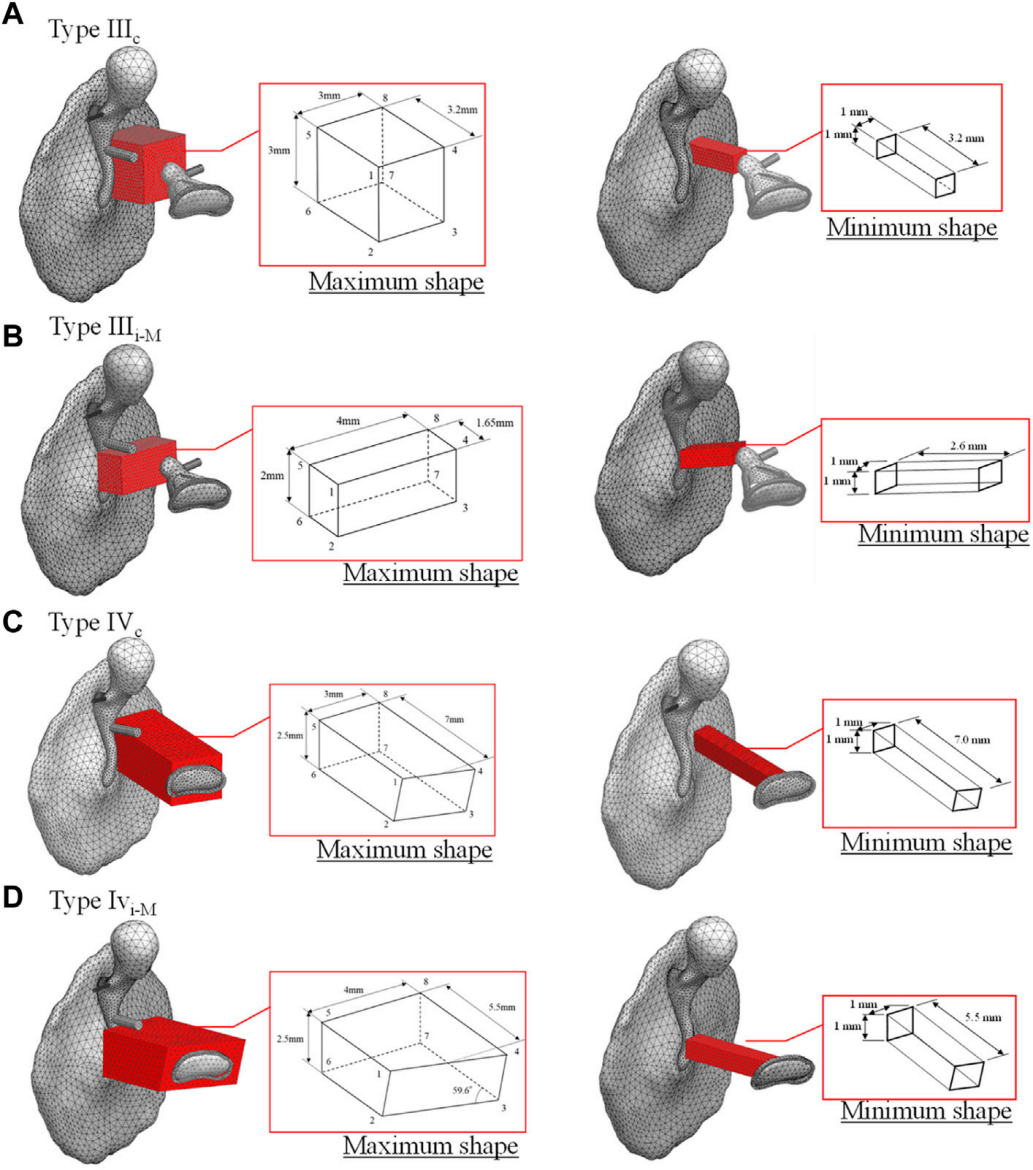
Original shape of tympanoplasty model types **(A)** III_c_, **(B)** III_i-M_, **(C)** IV_c_, and **(D)** IV_i-M_, respectively.

The analytical model for calculating the performance of an AO consists of the TM, ossicles, tendons, ligaments, and artificial prosthesis. As in the healthy model, the models including the artificial prosthesis were discretized with tetrahedral quadratic and linear tetrahedral elements for the vibration and acoustic elements, respectively. The physical properties of the artificial prosthesis are the same as those of the ossicles for Types III_c_ and III_i-M_ as shown in Table 1, because the artificial prosthesis is composed of the remaining ear ossicles. The physical properties of Types IV_c_ and IV_i-M_ were given assuming that they are composed of ear cartilage, as shown in Table 3. It should be noted that the physical properties of both of these materials, especially their Young’s modulus, are largely different and have a significant influence on the results obtained in this study. This is discussed in the Results and Discussion section. Because an otolaryngologist needs to manually process millimeter-scale autologous tissues, such as the incus and tragus cartilage, into an appropriate artificial prosthesis during tympanoplasty, we assumed a relatively simple processing and incorporated all reference shapes as prisms in the model to make the cross section of the prisms parallel to the TM surface. The reference shape and shape change pattern for each type of reconstruction model are shown in Figure 2. In these figures, the maximum and minimum shapes are defined for each type, and the acoustic transmission characteristics of columnar shapes with sizes intermediate between these two were controlled and evaluated. The modeling scheme of these columnar shapes is discussed in the appendices.

**TABLE 3:** Material properties for the incus and tragus cartilage used for the simulation of the Type III and IV models.

Material	Density (kg/m3)	Young’s modulus (MPa)	Poisson’s ratio (−)	Loss factor (−)
Incus	2.15×10^3^	1.20×10^4^	0.3	0.01
Tragus cartilage	1.10×10^3^	4		0.5

### Method of updating AO shapes

**FIGURE 3:**
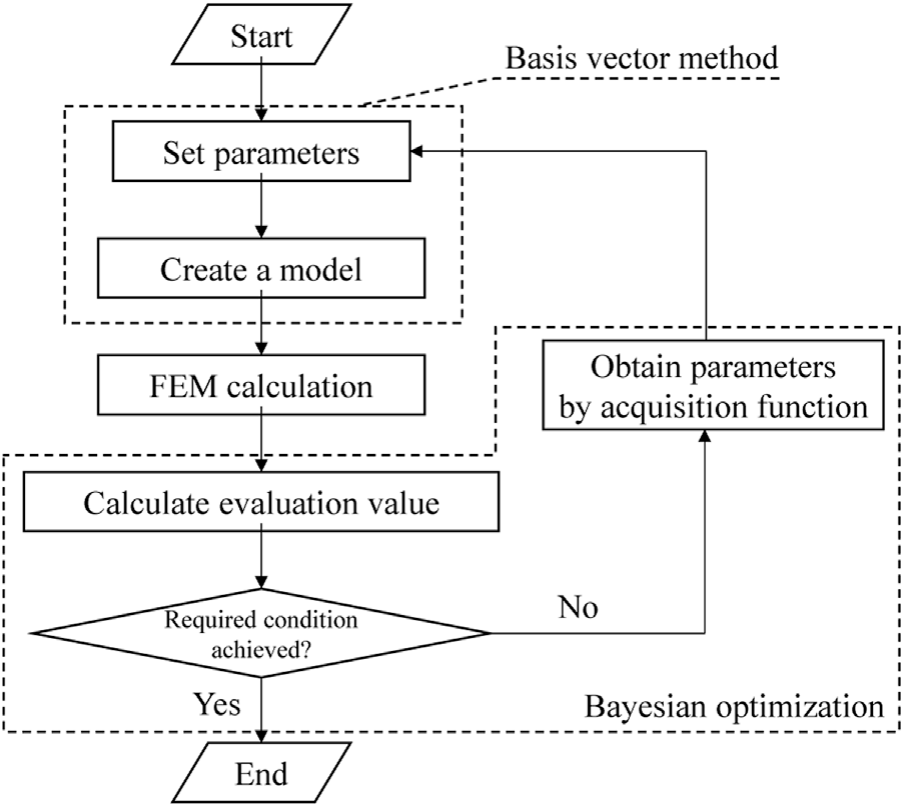
Flowchart of the updating calculation by the combined BVM-BO method.

Figure 3 shows a flowchart of the current study. Note that the detailed methodologies of BO and BVM are described in more detail in the appendices. The discrete FEM model was first created from the design variables output by the BVM. After that, the evaluation value, which was set as the hearing level in this study, was calculated from the intracochlear SPL. Then, if the required condition was not achieved yet, the design variable of the next step was specified from the acquisition function obtained by BO. The updated design variable was used to create a new FEM, and the above procedure was repeated. Here GPyOpt, a software library in Python, was used for the updating calculation by BO.

### Evaluation of vibroacoustic transmission characteristics

The simulation results of this study were evaluated as follows. In this study, the investigation described in the Results and Discussion section consists of three parts: the first part for validation of the intact middle-ear model, the second part for the effect of the AO shape on the frequency characteristics, and the third part for the detailed effect of the AO shape on hearing level. The first and second parts are discussed through detailed results of frequency responses of sound pressure inside the cochlea and displacements on the TM and footplate of the stapes, whereas the third part is discussed through a single-number quantity to simply investigate the relationship between the AO shape and the acoustic transmission performance. Thus, in this study, the average hearing level was adopted as the indicator of acoustic transmission performance. The hearing level measured by pure-tone audiometry is calculated as the level difference between the minimum audible level of a normal subject and the subject’s minimum audible level. Here, the average hearing level was calculated by averaging the hearing level values in three frequency bands of 500, 1,000, and 2,000 Hz (Tanaka, 2018). The hearing level was obtained by calculating the level difference between the intracochlear SPLs obtained in the conditions with the normal middle-ear model and the middle-ear model with an AO installed. The intracochlear SPL $p_{intracoch}$ was calculated as follows. First, the displacement at each discrete node on the stapes footplate was calculated by FEM, and the average displacement was calculated by averaging all the values. The intracochlear SPL $p_{intracoch}$ was calculated by substituting this average displacement into the following equation:

$$P_{intracoch}=20\log_{10}\frac{2\pi fVZ_{c}}{P_{0}},$$ (2)

where *f* is frequency, *V* is the volume displacement of the stapes footplate $\left(V=XS\right)$ , *S* is the area of the stapes footplate $\left(S=3.6\;{mm}^{2}\right)$ , *X* is the mean displacement of the stapes footplate, $Z_{intracoch}$ is the acoustic impedance of the intracochlear sound field $\left(Z_{intracoch}=D/S^{2}\right)$ , *D* is the viscous damping coefficient of the damper set on the inner ear side of the stapes footplate $\left(D=0.62\;Ns/m\right)$ , and *p*_0_ is the reference sound pressure $\left(p_{0}=2\times 10^{-5}\;Pa\right)$ . In the one-octave band centered on the frequencies 500, 1,000, and 2,000 Hz, the frequencies at which each frequency band was divided into four parts in a constant ratio were selected, the intracochlear SPLs at the 12 frequencies in total were calculated, and the average hearing level was calculated according to the method described above.

## Results and discussion

### Validation of the FEM results

**FIGURE 4:**
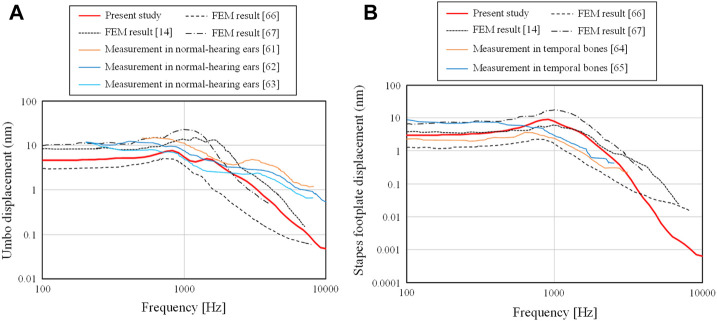
Comparison of the **(A)** displacements of the TM obtained at R_TM,u_ and **(B)** those of the footplate of the stapes obtained on S_foot_ with measurement and calculation.

To validate the FEM models of the intact middle ear, two types of displacements on the TM and footplate of the stapes were simulated. To evaluate the displacement of the TM, we used the displacements at $R_{TM,u}$ (Figure 1E). In contrast, for the displacement of the stapes footplate, we used the average vertical displacement obtained at the nodes over the entire area $S_{foot}$ of the footplate. The results are shown in Figures 4A, B, respectively. The frequency response was calculated by the FEM for a total of 100 frequencies equally distributed on the logarithmic axis in the frequency range of 100–10 kHz. Both Figures 4A, 1B also show reference values from previous literature measured *in vivo* and using the temporal bone (Gyo et al., 1987; Goode et al., 1996; Rodriguez et al., 1997; Huber et al., 2001; Nakajima et al., 2004), as well as previous simulation results using the FEM (Koike et al., 2002; Lee et al., 2009; Lee, 2020). Note that all of these results evaluate the displacements when a sound pressure of 80 dB SPL is applied on the TM surface.

First, the displacements obtained from the current FEM model were on the order of nanometers, which is consistent with the order of displacements obtained from experimental and analytical results in previous studies. The results obtained in this study are relatively flat from 100– 1 kHz for both the displacements at the umbo and footplate, but in the frequency range above 1 kHz, a slope-like decrease is observed, and this trend is similar to that observed in other experimental and analytical results.

In Figure 4A, a slight peak is observed in all results at the frequency where the displacement begins to decrease. However, the experimental results show no clear peaks and a smooth decay trend as the frequency increases. The current results show a characteristic with two moderate peaks and a smooth decay at higher frequencies, which indicate intermediate characteristics between the previous numerical and experimental results. The slope of the current study in the high-frequency band shows a steeper slope than that of the frequency characteristics of the previous experimental results, while they show almost the same degree of slope as the previous numerical results. The reason for this is currently not clear, but the loss coefficient assigned in the numerical analysis is a uniform value regardless of frequency. In real phenomena, the vibration damping caused in the higher frequency band may be slightly different from that in the band at around 1 kHz. Further studies are expected to be conducted on the detailed physical parameters of the entire ossicular chain.

In Figure 4B, the current FEM results and the previous numerical and experimental results show relatively similar trends, although the referenced experimental results are for frequencies only up to about 3 kHz. In the experimental results referred to in this figure, there is a slight peak around 1 kHz, which is not seen in Figure 4A. The FEM results also show a peak at a similar frequency, which confirms that the FEM models used in this study provide reasonable numerical results of the vibration characteristics of not only the TM but also the ossicular chain in the middle ear.

**FIGURE 5:**
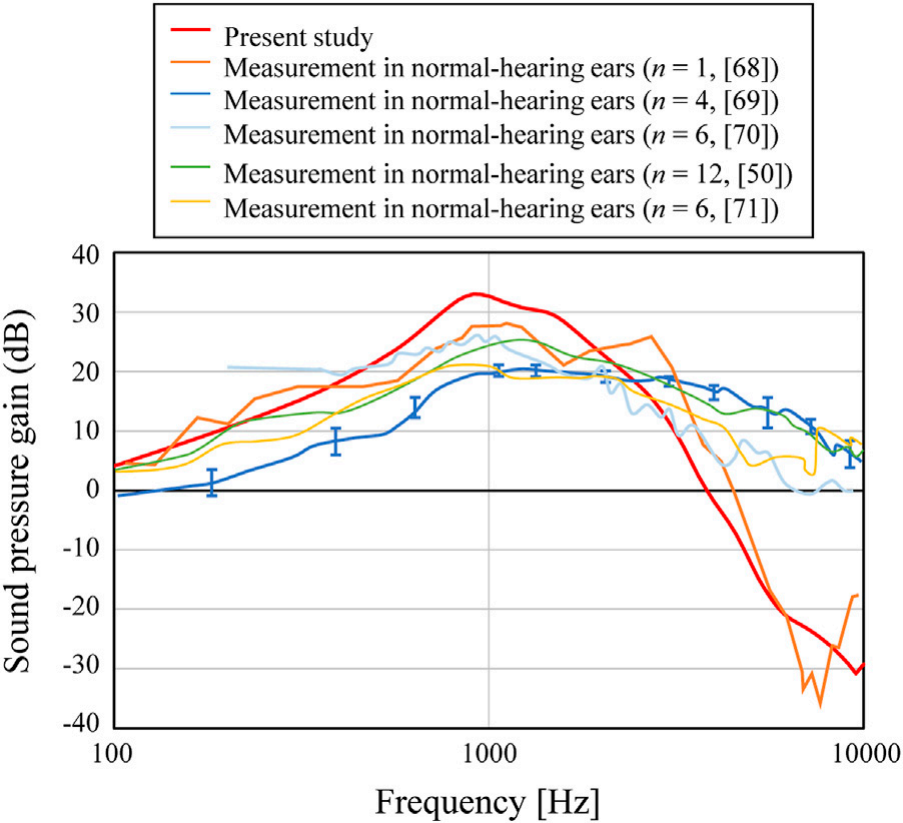
Comparison of the middle-ear pressure gain with the measured results.

Next, the intracochlear sound pressure level (SPL) is shown in Figure 5 in comparison with the previous results (Hüttenbrink and Hudde, 1994; Kurokawa and Goode, 1995; Puria et al., 1997; Aibara et al., 2001; Nakajima et al., 2009) obtained by actual measurement. In these previous results, a wide range of trends among the measured results can be seen. For example, the results by Puria et al. (1997) show a fairly flat frequency response, while the results by Hüttenbrink and Hudde (Hüttenbrink and Hudde, 1994) show a moderate peak from 1–3 kHz and a steep slope at higher frequencies. The current FEM results are rather similar to the results of (Hüttenbrink and Hudde, 1994), and differ from those of Puria et al. (1997), where the frequency trend increases up to higher frequency and a clear slope is not seen. The other three results (Kurokawa and Goode, 1995; Aibara et al., 2001; Nakajima et al., 2009) have a peak at the lowest frequency around 1 kHz and a relatively clear slope above 1 kHz, and their characteristics also differ from those of Puria et al. (1997). It can be considered that the current FEM results indicate characteristics similar to the measurements other than Puria et al. (1997) from the viewpoint of the frequency characteristics, with a peak at 1 kHz and a clear slope above 1 kHz.

Although the trends of the FEM results indicate frequency trends similar to each other, the numerical results also showed a variety of amplitude levels with a difference of about >10 dB at most. At low and medium frequencies below about 3 kHz in Figure 5, there is a difference of up to about 10 dB. In the existing literature (Greef et al., 2017), the influence of the physical properties given in FEM simulation of the acoustic transmission characteristics of the middle ear on the obtained results was examined, and it has been shown that the simulated results are largely affected by the individual geometric models, as well as by the physical properties. Although the numerical results presented in this study differ in some details from previous calculations and experimental results, the overall trend of the frequency characteristics is considered to be similar to that shown by previous studies. The aforementioned study verified the accuracy of the numerical simulation required to numerically verify the effect of the AO shape on acoustic transmission characteristics, which is the goal of the current study.

### Effect of the AO shape

**FIGURE 6:**
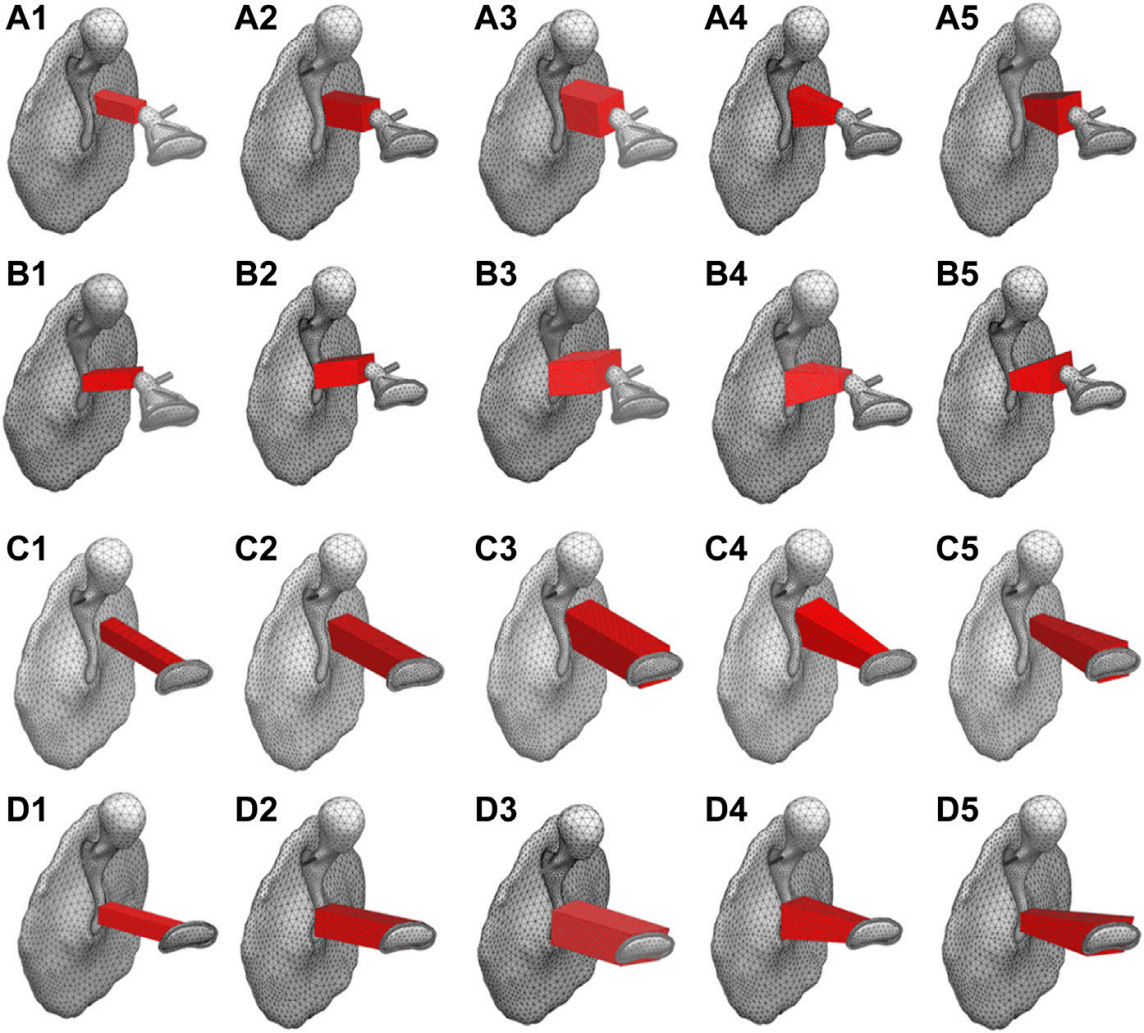
Investigated AO shapes of **(A1 -A5)** Type III_c_ **(B1 -B5)** Type III_i-M_ **(C1 -C5)** Type IV_c_, and **(D1 -D5)** Type IV_i-M_.

**FIGURE 7:**
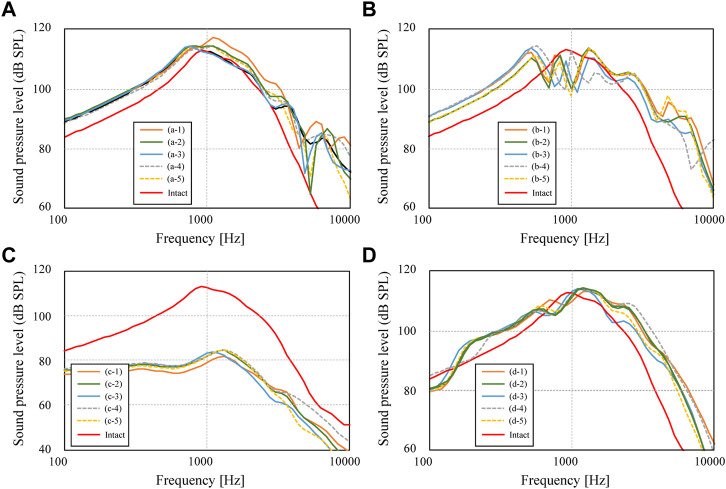
Sound pressure levels inside cochlea for conditions of **(A)** Type III_c_, **(B)** Type III_i-M_, **(C)** Type IV_c_, and **(D)** Type IV_i-M_.

**FIGURE 8:**
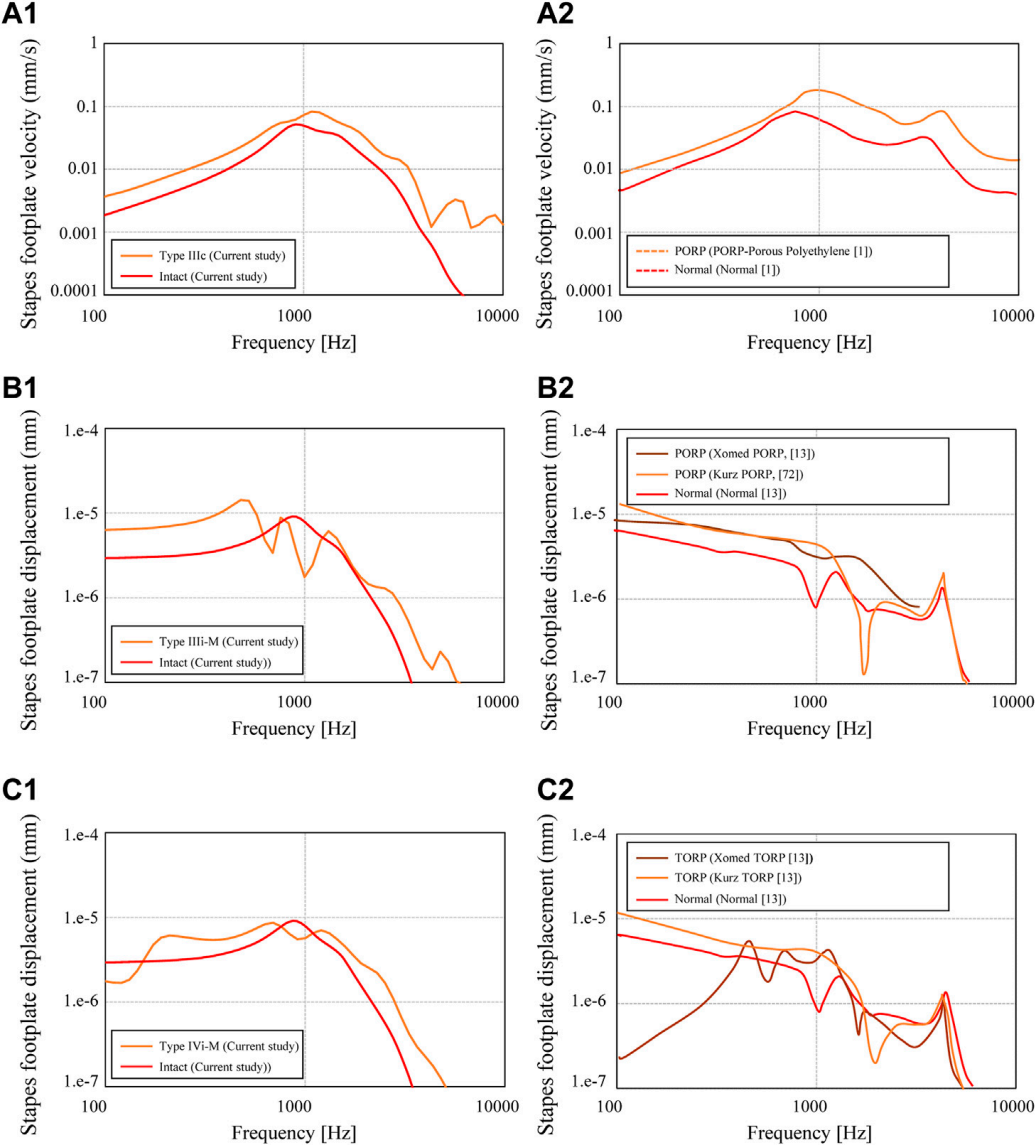
Stapes footplate displacement calculated for the conditions of **(A1)** Type III_c_ (Figure 8A, “Minimum shape”) **(B1)** Type III_i-M_ (Figure 8B, “Minimum shape”), and **(C1)** Type IV_i-M_ (Figure 8A, “Minimum shape,“; Figure 8D, “Minimum shape”). The referenced results are indicated in **(A2, B2, C2)** for each of the abovementioned types.

First, among the shapes that exist between the maximum and minimum shapes shown in Figure 2, a total of five shapes were generated for each AO type: three columnar AO shapes with different cross-sectional areas, and two truncated square pyramids with wide or narrow areas on the tympanic side. The acoustic transmission characteristics of these AOs were simulated and compared. The shapes of all 20 AOs are shown in Figure 6. In this figure, the columnar shape has a cross section of 1 mm × 1 mm (a-1, b-1, c-1, d-1, e-1), 1.5 mm × 1.5 mm (a-2, b-2, c-2, d-2, e-2), and 2 mm × 2 mm (a-3, b-3, c-3, d-3, e-3). The shapes of the truncated square pyramid are as follows: a condition in which the surface connected to the TM has a large area (a-4, b-4, c-4, d-4), and a condition in which the surface connected to the TM has a narrow area (a-5, b-5, c-5, d-5). In all conditions, the volumes of a/b/c/d-4 and a/b/c/d-5 are equivalent to the corresponding a/b/c/d-2 shapes. In other words, each of these models has an equivalent average cross-sectional area. The results of the simulation are shown in Figure 7. First, as shown in Figure 7A, which indicates the results of Type III_c_, as the cross-sectional area of the AO becomes narrower from a-3 to a-1, the acoustic transmission characteristics in the frequency band above 1 kHz improve. In contrast, both truncated square pyramids a-4 and a-5 show trends similar to that of a-2, which has an average cross-sectional area similar to that of a-4 and a-5. In addition, the effect of the change among the pyramid-like shapes is also minor; Figure 7B shows dips in almost all conditions at frequencies around 1 kHz. However, there is an increase in the SPL in the frequency range lower than 1 kHz. Conditions b-1 and b-2 indicate almost the same characteristics, while b-3 indicates slightly lower SPLs in all frequency ranges. Conditions b-4 and b-5 vary with frequency, with the former showing a higher value in the low-frequency range, while the latter shows a higher value in the high-frequency range; Figure 7C shows that the SPLs of all AO conditions indicate lower values in all frequency bands compared to the intact condition. The relative relationships among the five conditions are similar to those described in Figure 7B. The values of c-4 and c-5 are comparable up to about 3 kHz for both conditions, while slightly higher for c-4 above that frequency. The reason of the very low SPLs only in this condition is that Type IV uses the physical properties of cartilage for the AO, which has a relatively low Young’s modulus, so the AO is easily bent and deformed. In addition, another reason is that the AO of this condition is in contact with the quite soft TM instead of the hard and vibration-transmissive manubrium of the malleus. In Figure 7D, similar relative relationships as in Figure 7C among the five AO conditions are found. Although the shape of each AO and the method of connection to the TM and stapes differed among the four AO types, there was a decrease in SPL under the conditions with a relatively large cross-sectional area. In addition, differences in the AO geometry appeared to affect the SPL in the case of Type III_i-M_, but not in the other types. This is because, only in this Type III_i-M_, both ends of the AO are connected to relatively hard materials, the manubrium of the malleus and the head of the stapes, and Type III has a Young’s modulus value equivalent to that of the ear ossicles, which is relatively higher. As a result, Type III_i-M_ has a more complex peak and dip shape due to the increased stiffness of the entire ossicular chain. In contrast, the AO types other than Type III_i-M_ were connected to the TM or footplate of the stapes, which are relatively soft, so the effect of the shape was considered to be less pronounced. To confirm the validity of the simulation, these results were compared to previous studies (Ferris and Prendergast, 2000; Kelly et al., 2003; Jiang et al., 2017) that measured the acoustic transmission characteristics of the middle ear with similar artificial prostheses. The results are shown in Figure 8. Note that the shape of the AO in each of the papers referenced in the following comparison is not perfectly equivalent to the AO adopted in this paper, resulting in a qualitative comparison.

First, the simulated results for the slenderest geometry of Type III_c_ (Figure 6(a-1)) and the measured results for the PORP by Jian et al. (2017), which is similar to the Type III_c_ geometry, are comparatively shown in Figure 8(A-1) and Figure 10 (A-2), respectively. The results of the displacement of the footplate of the stapes obtained in this study show higher acoustic transmission characteristics for Type III_c_ than for the intact condition, while Figure 8(A-2) shows that the acoustic transmission characteristics are also improved over the entire frequency range. Next, Figure 8(A-2) and (B-2) show the results for the slenderest Type III_i-M_ (Figure 6(b-1)), the Xomed PORP by Kelly et al. (2003), and the Kurz PORP by Ferris and Prendergast (2000). It can be seen that the results of this study show that, below 1 kHz, where the dip occurs, Type III_i-M_ shows higher sound propagation characteristics than those of the intact conditions, and even at higher frequencies, Type III_i-M_ also shows slightly higher values than those of the intact condition. Figure 8(B-2) shows that the Xomed PORP results are higher than those of the normal condition in the frequency band below around 3 kHz. In addition, the Kurz PORP has a sharp dip at around 2 kHz and the values for frequencies lower than 2 kHz indicate higher values in the Kurz PORP than in the normal condition. This suggests that the model in this reference (Kelly et al., 2003) also has a sharp dip due to increased stiffness of the ossicular chain. In contrast, the Xomed PORP results in this figure show just a moderate dip, which is noticeably not as sharp as in the Kurz results. This contrast is because the Kurz PORP is made of hydroxyapatite (Young’s modulus: 155 GPa) and Xomed PORP is made of titanium (Young’s modulus: 116 GPa). The latter is less rigid, so although no noticeable dip is observed in the frequency range shown in Figure 8(B-2), it is possible that a dip is observed at frequencies higher than those of the published range.

Next, the results of the FEM model for the slenderest shape among Type IV_i-M_ (Figure 6(d-1)) are shown in Figure 8(C-1), whereas the measured results for the Xomed and Kurz TORP by Kelly et al. (2003), which are similar to the model of the current study, are shown in Figure 8(C-2). The results of the current Type IV_i-M_ show higher acoustic transmission characteristics than in the intact conditions, except in the band around 1 kHz, which has a dip. The overall trend is not very different from the relationship seen in Figure 8(B-1). The relationship between the normal condition and the Kurz TORP seen in Figure 8(C-2) is similar to the relationship between the normal one and the Kurz PORP seen in Figure 8(B-2), showing that the relationship between the normal and reconstructed geometry is different between the frequency ranges lower and higher than 2 kHz. At frequencies lower than 2 kHz, the TORP condition indicates a higher value than that of the intact condition, while at frequencies higher than 2 kHz, the TORP condition indicates a lower value than that of the intact one. A similar inversion is also observed in the Xomed TORP, with a dip around 2 kHz.

To summarize, in comparison with the results of the previous studies mentioned above, we were not able to perform a comparative verification under perfectly matched conditions, but a similarity of the acoustic transmission trend between the intact and AO conditions was confirmed. In the next section, the influence of AO geometry on acoustic transmission characteristics is parametrically discussed by using a combined BO-BVM approach.

### Improvement of acoustic transmission characteristics through optimization

**FIGURE 9:**
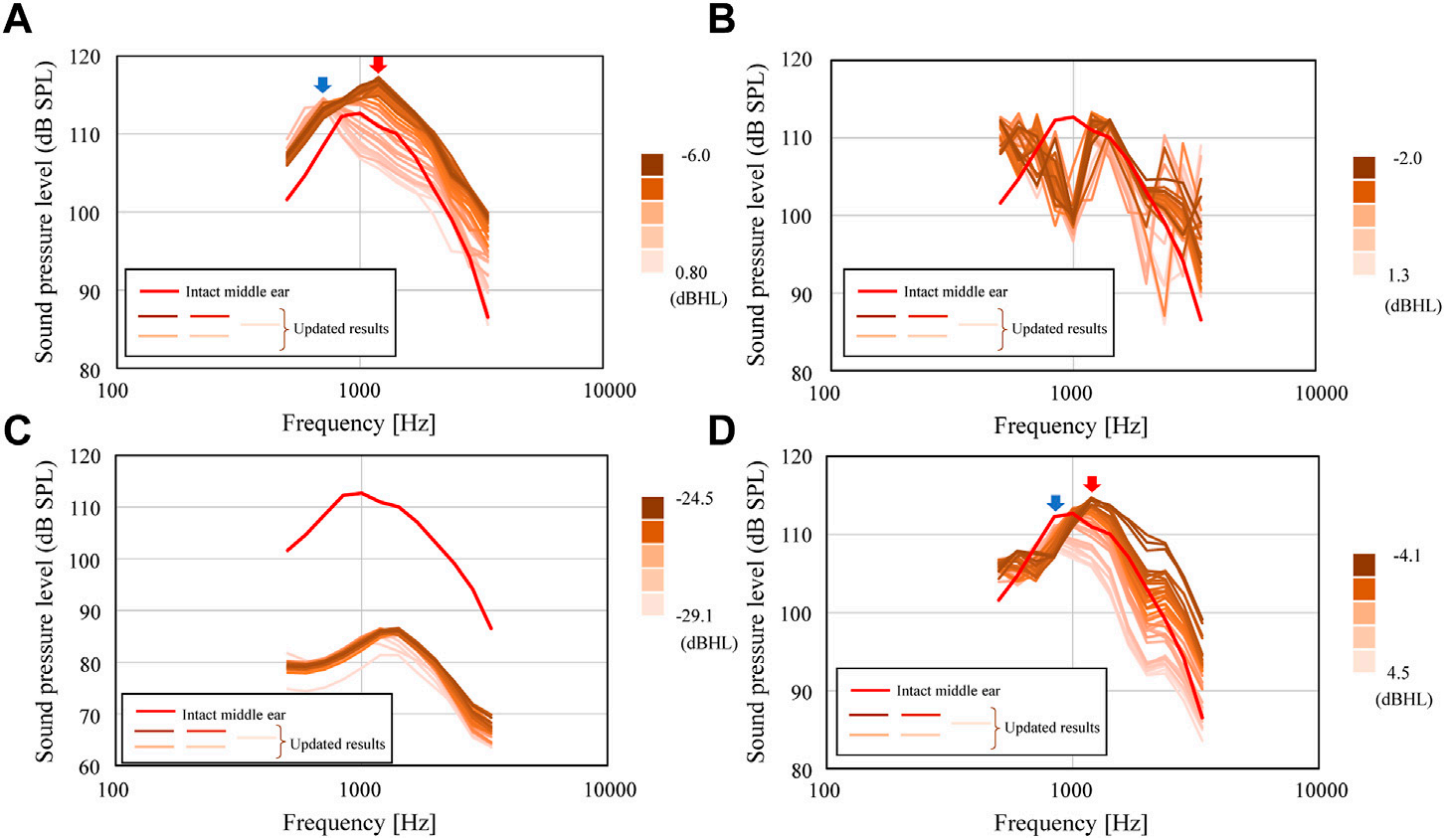
Updated SPLs inside the cochlea for the conditions of **(A)** Type III_c_, **(B)** Type III_i-M_, **(C)** Type IV_c_, and **(D)** Type IV_i-M_.

**FIGURE 10:**
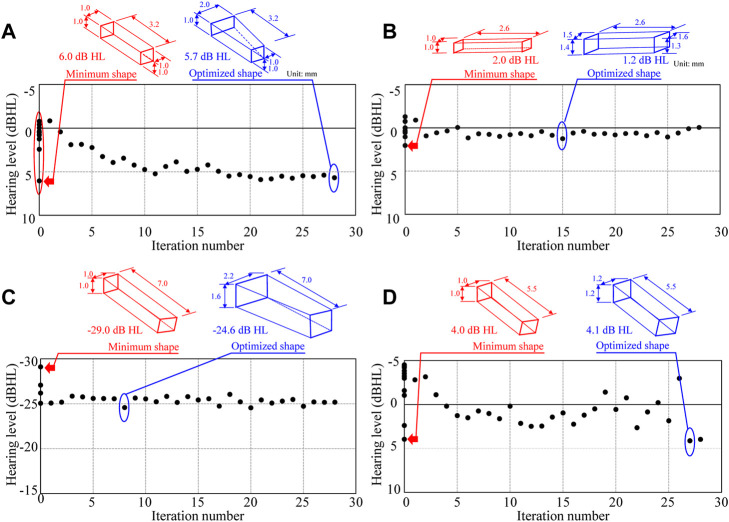
History of updated hearing levels in the updating scheme for the conditions of **(A)** Type III_c_, **(B)** Type III_i-M_, **(C)** Type IV_c_, and **(D)** Type IV_i-M_.

The results of the updating calculation performed using the combined BO-BVM method are shown in Figure 9. The number of iterations for this updating calculation was set to 28 to allow all the calculations to be completed in half a day, and the changes in AO shapes and acoustic transmission characteristics obtained during the iterations are discussed. As a result, the possible shapes of an AO were efficiently explored and the impact of AO shapes on the hearing level was investigated as described below. In this updating method, the information on an AO shape output by BO is input to BVM to output the 3D shape of the AO, and the hearing level achieved by the shape is calculated and obtained by the FEM. Figure 10 shows the updated results of the hearing level for 28 updating calculations. In Figures 10A–D, the optimized shapes of the AOs are additionally illustrated as well as the minimum shapes of the AO is indicated as a reference. In Figure 10A, 11 points are plotted at iteration number 0 surrounded by the red ellipse. These are the hearing levels for 11 types of AO set as an initial condition of BO, which were preliminarily obtained by performing FEM analysis. The 11 types of AO were as follows: three conditions of quadratic prisms with cross-sectional areas of 1.0 mm × 1.0 mm, 1.5 mm × 1.5 mm, and 2.0 mm × 2.0 mm, and eight conditions with each of the eight components from *α*_1_ to *α*_8_ in the eight vectors (b) to (i), as shown in Supplementary Appendix Figure A1, trimmed by 1 mm each. Note that this initial condition is necessary to calculate the prior probability in BO, so in this study, the 11 shapes described above were used as the initial conditions. Future work is intended to provide beneficial guidance on what initial conditions should be used.

First, the results of the iterative calculations for Type III_c_ in Figure 9A show that the hearing level ranges from −6.0 to 0.8 dB HL, depending on the shape of the AO, with a difference of more than 6 dB HL. The variation of the hearing level is due to the large variation of the frequency characteristics around 1 kHz, which is important in the evaluation of hearing level. In Figure 10A, the blue and red arrows indicate the range where the aforementioned shift of the peak around 1 kHz is pronounced, indicating that the frequency of the peak changes significantly when the AO shape changes, which in turn affects the sound transmission characteristics around 1 kHz. The change in the output values of the hearing level in this iterative calculation (Figure 10A) shows that the hearing level obtained for the 11 conditions initially given as initial conditions are distributed from a maximum value of 6.0 dB HL to a minimum value of −0.9 dB HL. The condition with the highest value of 6.0 dB HL was the most elongated quadratic prism shape with a cross section of 1 mm × 1 mm. Then, the iterative calculations resulted in a gradual increase in the hearing level output from each step of the calculations, with the hearing levels ranging from a minimum value of −0.9 dB HL to a maximum value of 5.9 dB HL. Here, the 28th result showed a maximum hearing level of 5.9 dB HL, while the first result showed a minimum hearing level of −0.8 dB HL. The dimensions of the AO shape obtained in the 28th iteration of the calculations formed nearly quadratic prism with a cross sectional area of 1 mm × 1 mm, where only *α*_5_ among the coefficients from *α*_1_ to *α*_8_ shown in Supplementary Appendix Figure A1 is 0 and all other seven parameters are 1.0. Therefore, Type III_c_ is considered to have been explored relatively evenly throughout the range of hearing levels from 6.0 to −0.9 dB HL, although it did not produce a hearing level higher than the 6.0 dB HL obtained under the initial condition. The results of Figure 9B for Type III_i-M_ show a sharp dip around 1 kHz, as already shown in Figure 7B. Although this dip is seen in all reconstructed ossicles, the peak and dip characteristics around the frequencies above and below it, as well as around 3 kHz, appear to fluctuate wildly. This may be due to the fact that Young’s modulus is given a high value similar to that of the ear ossicles under these conditions, and that the stiffness of the entire ossicular chain is increased by the connection of both ends of the AO to the ossicles. The results of the subsequent iterative calculations in Figure 9C for Type IV_c_ do not show much change in the sound transmission characteristics. As discussed in the next section, although the geometry of the AO changed quite dramatically over the 28 update calculations under these conditions, its acoustic transmission characteristics did not change much. Thus, as seen in Figure 10C, the hearing levels also changed little. In this condition, as mentioned above, the stiffness of the AO is lower than that in the other conditions, and the piston vibration of the TM is not efficiently transmitted to the stapes footplate, so it is assumed that the change in shape had only a minor effect on the acoustic transmission characteristics. Finally, the results of the iterative calculations shown in Figure 9D for Type IV_i-M_ also show that, as in Type III_c_, as the shape of the AO changes, the peak frequency changes gradually, as indicated by the blue and red arrows, which consequently affects the acoustic transmission characteristics around 1 kHz. In particular, as the resonance frequency approaches the frequency indicated by red arrows, the acoustic transmission characteristics around 1 kHz also improve as described in detail in the next section.

Looking at the results of these iterative simulation, it can be seen in Figure 9 that for Types (a) III_c_ and (c) IV_i-M_, the resonance frequency occurring around 1 kHz moves to a lower frequency as the size of AO increases, as will be discussed in the next section. As a result, the acoustic transmission characteristics in the frequency domain above 1 kHz are affected and reduced, resulting in worse hearing levels. On the other hand, in Type (b) III_i-M_, when the volume of the AO changes, the frequencies of the peaks and dips in the high frequency appear to be affected by the random fluctuations. These results suggest that the acoustic transmission characteristics in the high-frequency domain are significantly affected by changes in the sizes of the AO to a large extent.

The results given in this section suggest that the dimensions of the AO shapes have an influence on the acoustic transmission characteristics. In the next section, the relationship between the AO shape and the hearing level is discussed further quantitatively.

### Effect of the reconstructed middle-ear volume

**FIGURE 11:**
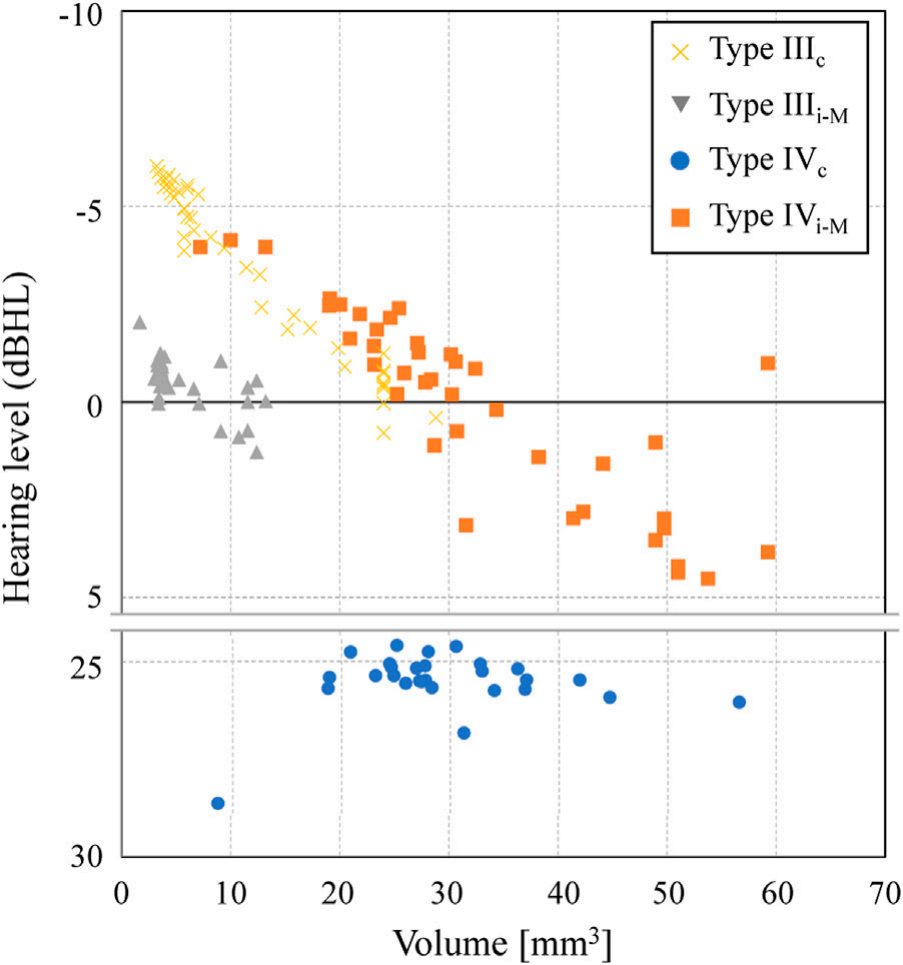
Effect of the AO volume on the hearing level in each type of middle-ear reconstruction.

The acoustic transmission characteristics of each type obtained in the updating calculation of the previous section are shown in Figure 11 as the relationship between the volume of each type of reconstructed shape and the hearing level. In Types III_c_ and IV_i-M_, the acoustic transmission characteristics improve as the volume decreases. These are AO types with different installation conditions from each other, because the AO in these types is connected to the TM (III_c_) or to the petiole of the tarsus (IV_i-M_). However, they both show comparable values, especially around 20–30 mm^3^, whereas Type IV_i-M_ lacks the superstructure of the stapes. The Type III_i-M_ contrastingly indicates values about 5 dB HL lower than those of Type III_c_, but even within the Type III_i-M_ variations, the hearing levels have a slight tendency to increase with decreasing volume. Type IV_c_, while not showing a definite trend because of its low hearing level, also shows a slight linear increase in hearing level with decreasing volume, albeit at a slower rate than that of the other types. While the geometry was different from the AO in our study, the effect of AO diameter has been discussed in previous studies. Grolman et al. (1997) and Sennaroğlu et al. (2001) concluded that a thicker AO has better acoustic transmission characteristics, although they studied very thin members less than 1 mm, while Shabana et al. (1999) concluded that the effect of thickness is negligible. In contrast, although all the shapes handled in this study are columnar with side lengths of 1 mm or more to ensure ease of making by otolaryngologists, the AOs with side lengths closer to 1 mm showed higher acoustic transmission characteristics. Although this study did not examine the effect of AOs with dimensions thinner than 1 mm, as in the various studies mentioned above, by expanding the search for updating calculations to a wider range, consistency with the trends seen in these previous studies can be confirmed, which may lead to more useful findings in the future.

### Limitations

The discussion in the current study is based on the FEM, and the accuracy of the simulation was verified through comparison with currently available examples of previous studies. The findings regarding the influence of AO shapes are discussed in the previous section. However, the prediction accuracy of the FEM, that is, its validity in various models that depend on the shape of the ossicular chain, which varies from individual to individual, and detailed effects by each of the biological parts in the middle ear such as tendons and ligaments, has not yet been verified. In addition to the effects of above factors, the physical parameters set in the numerical analysis are not yet in a situation to use values that are fully consistent with practical phenomena. In particular, the comparison between the former experimental and current simulative results has partially indicated characteristic differences. Shi et al. (2023) have examined modelling methods that can better explain the phenomenon by comparing the results of FEM analyses of middle ear acoustic propagation using various damping models. While in order to focus on the effect of the shape of the AO on the acoustic propagation characteristics, frequency-independent loss factors were simply assigned in the current study, there is a need to deeply examine the effect of damping models to obtain results that contribute more to the actual hearing. Furthermore, in actual surgery, the position of the tympanic membrane and the ossicles may be deviated from intact state in case of diseases such as chronic otitis media or middle ear pearls. Such cases may show different acoustic transmission characteristics from the current middle-ear model. As a future work, pathologies that are more likely to occur in real cases, such as tympanic membrane retraction or stapes inclination, should be added to the modelling.

The updating scheme used in this study, which combines BO and BVM, is expected to be ultimately used to determine the optimal solution for various conditions, including the shape of the AO and its physical properties, but the optimization itself has not yet been examined in this study. While this study provided useful information for intraoperative ossicular reconstruction using autologous bone or cartilage, research on highly flexible design approach on the optimized prostheses has also been carried out (Milazzo et al., 2020; Dargah et al., 2023). In the future, a comparative study of the differences between the proposed methods for prosthesis optimization should be carried out. Through such a series of studies, further studies are desired to expand the application range of the FEM and develop an optimal AO design method that matches the conditions in the middle ear of each individual patient based on the optimization method, as has been pointed out in previous studies (Kamrava and Roehm, 2017).

## Conclusion

The current study investigated the effect of the shapes of reconstructed AOs on the acoustic transmission through the middle ear by a numerical approach using the FEM. First, a numerical study using the FEM was conducted by efficiently choosing the calculation parameters using BO. Then, a FEM simulation was carried out to investigate the frequency response through the middle ear, and the numerical results were validated through comparison with previous experimental and numerical results. Second, the middle-ear reconstruction models of Types III and IV were considered as the target models, and the effect of the AO shapes on the acoustic transmission characteristics was obtained by an updating calculation based on the combined BO-BVM method. Based on these schemes, the vibroacoustic mechanism of the acoustic transmission through artificial prostheses with various simple shapes was clarified. The results specifically indicated that especially the volume of the AO prosthesis has a great influence on the numerically obtained hearing levels.

## Data Availability

The original contributions presented in the study are included in the article/Supplementary Material, further inquiries can be directed to the corresponding author.
