# Supplementary material for: Mechanical effect of reconstructed shapes of autologous ossicles on middle ear acoustic transmission
Source: Front Bioeng Biotechnol. 2023 Jun 22;11:1204972. doi: 10.3389/fbioe.2023.1204972 (PMC10323686; doi:10.3389/fbioe.2023.1204972)
Supplement: Supplementary file 1 [file DataSheet1.PDF]

Appendices

Using BVM to model reconstructed ossicle shapes. BVM is a simple method for expressing patterns of shape change. In this method, the shape of the target object is defined by a superposition of the original shape and several basis shapes that are optimally weighted in the superposition. Specifically, the case of changing the shape of Type III_c [Figs. 10(a) and A.1] is described as follows. In Fig. A.1, the basic shape is first defined as shown in Fig. A.1(a). Then, the basis shapes are defined as the 3D shapes indicated by the black line in Figs. A.1(b) to (i). In these eight types of shapes, the position of each edge represented by a gray numeral with an apostrophe is arbitrarily changed, and these are superimposed to represent a new shape. Each shape is represented as a coordinate vector at the vertex of each shape, and is combined by multiplying the difference between the basis vector (a) and the j th basis shape vector [$j = 1, 2, \ldots, 8$ in this study, representing eight types from (b) to (i)] by the design variable α_j, as in the equations below. Note that because all the basis shapes used in this study have eight vertices each, the number of vertices i in the equation below is set to 8.

$$X_i = X_{\mathrm{org},i} + \sum_j \alpha_j \left(XB_{ij} - X_{\mathrm{org},i}\right), \qquad (A.1)$$

$$Y_i = Y_{\mathrm{org},i} + \sum_j \alpha_j \left(YB_{ij} - Y_{\mathrm{org},i}\right), \qquad (A.2)$$

$$Z_i = Z_{\mathrm{org},i} + \sum_j \alpha_j \left(ZB_{ij} - Z_{\mathrm{org},i}\right), \qquad (A.3)$$

where X_i, Y_i, and Z_i are vectors representing the coordinates of each vertex of the newly formed model; $X_{\mathrm{org},I}$, $Y_{\mathrm{org},i}$, and $Z_{\mathrm{org},i}$ are vectors representing the coordinates of the i th vertex of the original shape [Fig. A.1(a)]; XB_{ij}, YB_{ij}, and ZB_{ij} are vectors representing the coordinates of the j th basis shape [Figs. A.1(b) to A.1(i)]; and α_j represents the design variable for the j th basis shape [Figs. A.1(b) to A.1(i)]. Shape modifications in the Type III_c shape were performed as follows. For the shape modification, the minimum shape of the reconstructed ear ossicles was determined to be a prism with a cross-sectional area of 1 mm × 1 mm as shown in the right side of Fig. 10(a). Therefore, the cross-sectional area of the superposed shape is 1 mm × 1 mm when all the design variables α_j are at their minimum value of 0 [Fig. 10(a), right], while the cross-sectional area is 3 mm × 3 mm when all the design variables α_j are at their maximum value of 1.0 [Fig. 10(a), left].

The expressions of reconstructed shape typology other than those described above are shown below. The shape change pattern for $III_{i\text{-}M}$ is set to be the minimum shape with a cross-sectional area of 1 mm × 1 mm when all the design variables α_j have a minimum value of 0 [Fig. 10(b), right], while the maximum shape shown in Fig. 10(b) is obtained when all α_j have a maximum value of 1.0 [Fig. 10(b), left]. The shape change pattern of IV_c is set so that the minimum shape with a cross-sectional area of 1 mm × 1 mm [Fig. 10(c), right] is obtained when all α_j have a minimum value of 0, while the maximum shape shown on the left of Fig. 10(c) is obtained when all α_j have a maximum value of 1.0. The shape change pattern of $IV_{i\text{-}M}$ is set so that the minimum shape with a cross-sectional area of 1 mm × 1 mm [Fig. 10(d), right] is obtained when all α_j have a minimum value of 0, while the maximum shape shown on the left of Fig. 10(d) is obtained when all α_j have a maximum value of 1.0.

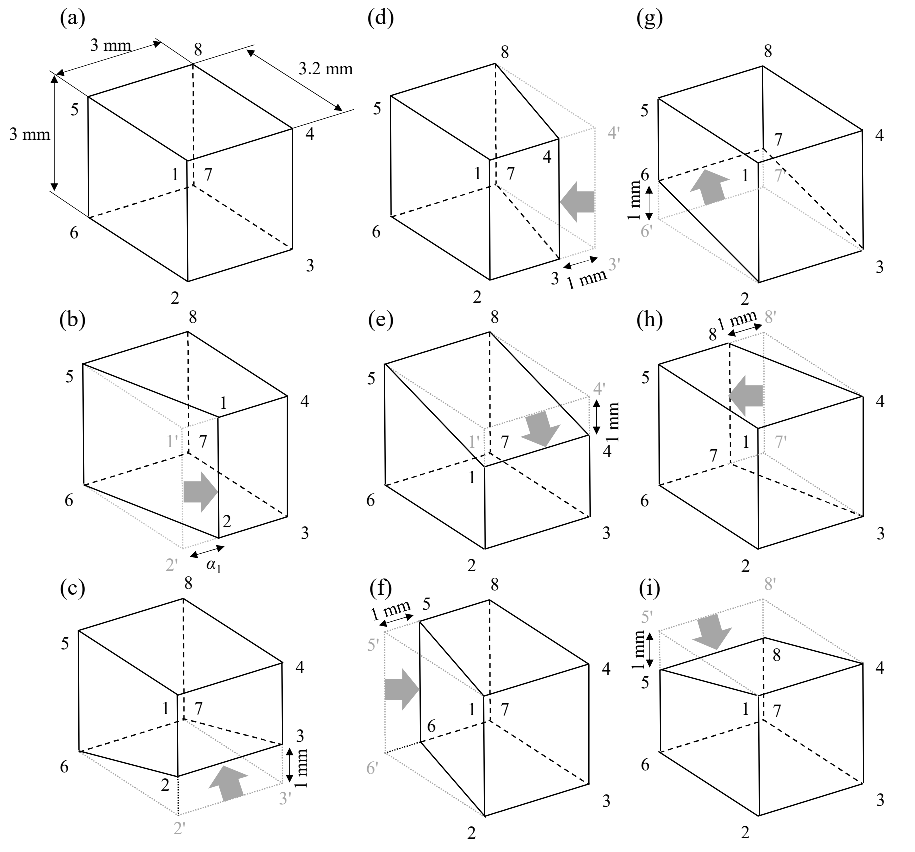

Fig. A.1. Example of original and basis shapes of Type III$_c$: (a) Original shape and (b)-(i) basis shapes.

Update calculation employing BO-BVM. The influence of the shape change on acoustic transmission characteristics was examined by using BVM, which can quantitatively describe the shape of the target object of optimization, and BO, which can efficiently find the optimal value for an unknown function. First, the search flow for the optimal solution by BO is shown in Fig. A.2. Assuming input value $\mathbf{x}$ and output value y, when N input and output data ($\mathbf{x}_1$, $\mathbf{y}_1$), …, ($\mathbf{x}_N$, $\mathbf{y}_N$) are obtained as a result of N experiments, the objective function $f(\mathbf{x})$ is obtained based on a Gaussian process in the form of an expected value and standard deviation of the posterior probability. Then, the input value $\mathbf{x}_{N+1}$ at the next step is determined where the acquisition function defined by combining the expected value and standard deviation can be maximized. In this search, the expected value that the next observation point $\mathbf{x}$ improves the current best function value τ is evaluated, and then the $\mathbf{x}$ with the largest expected value is set as the next evaluation point. This process is repeated to efficiently search for the optimal solution. Note that, in this study, the expected improvement (Jones et al., 1998) was used as the acquisition function.

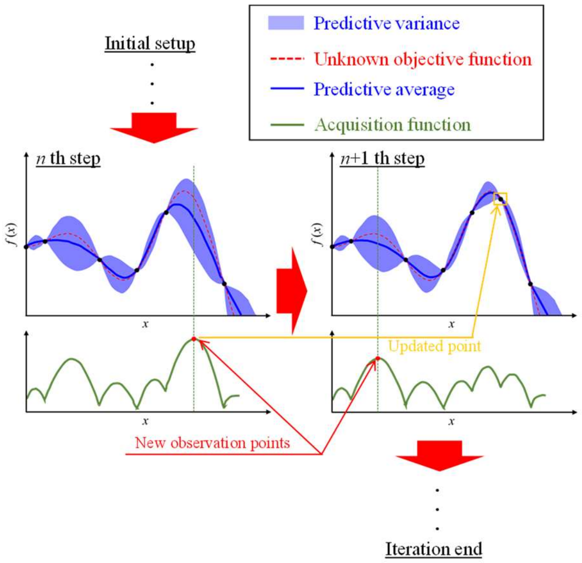

Fig. A.2. Flowchart the BO updating procedure.
